# COVID-19 in the Americas: Who’s Looking After Refugees and Migrants?

**DOI:** 10.5334/aogh.2915

**Published:** 2020-06-29

**Authors:** Maximo O. Brito

**Affiliations:** 1 University of Illinois at Chicago, College of Medicine, Chicago, US; 2 Jesse Brown VA Medical Center, Chicago, Illinois, US; 3 Department of Medicine Inclusion Council, University of Illinois at Chicago, Illinois, US

## Abstract

Several characteristics of refugee and migrant populations make them susceptible to acquire COVID-19. To fully understand the impact of COVID-19 on refugees and migrants in the Americas, it is important to consider the broader geopolitical context and appreciate the differences among migratory groups. There are three migrant groups in the Americas that are particularly susceptible to COVID-19: Central American migrants at the northern Mexico border, Venezuelans within South America, and Haitians in the Dominican Republic. Refugees and displaced migrants are the world’s collective responsibility, and thus, it would be imprudent to displace their care to resource constrained developing nations.

Refugees and displaced migrants are particularly vulnerable to infection with the SARS- CoV-2 virus and therefore present a substantial challenge to global pandemic mitigation efforts. The term *refugee* is used to describe people who leave the country of their nationality due to a well-founded fear of persecution and who are unable or unwilling to avail themselves of the protection of that country [1]. *Migrant* describes “persons who move away from their place of usual residence, whether within a country or across an international border, temporarily or permanently, and for a variety of reasons [2].”

Several characteristics of refugee and migrant populations make them susceptible to acquiring respiratory diseases such as COVID-19. First, refugees and migrants typically live in overcrowded and unsanitary shelters or multigenerational inner city housing, which impair their ability to adhere to proper hand hygiene as well as to maintain appropriate physical distance from each other. Second, they have poor access to healthcare services due to a lack of health insurance coverage and because of a pervasive fear of identification by law enforcement in their host country. Moreover, even when they utilize healthcare services, they often are unable to afford medications to treat underlying chronic diseases, like diabetes and hypertension, which provisionally appear to increase the severity of COVID-19 infection. Third, refugee and migrant populations are highly mobile, which increases the opportunities for close contact and exposure to other people affected with respiratory diseases such as COVID-19.

To fully understand the impact of COVID-19 on migrants and refugees in the Americas, it is important to consider the broader geopolitical context and appreciate the differences among migratory groups. Poverty, political instability, natural disasters and violence have shaped migration tendencies in the Americas over the past decade. There are three migratory currents in the Americas with relatively large terrestrial movements of persons: northbound migrations of Central American persons traveling through Mexico to reach the southern United States (US), the movement of Venezuelan nationals within South America, and the influx of Haitian migrants to the Dominican Republic.

Among these groups, the greatest potential for a humanitarian crisis to unfold during this pandemic involves the northward migration of Central Americans. According to the United Nations High Commissioner for Refugees (UNHCR) approximately half a million people irregularly cross the southern Mexican border every year to seek asylum or to begin the more than 1,000 mile journey to the southern US border. The large majority of these migrants come from Guatemala, El Salvador, and Honduras, which constitute the Northern Triangle of Central America [3]. Migrants who reach the US border either present themselves to US authorities for asylum or attempt to cross the border irregularly from a Mexican border town. US Customs and Border Protection reported nearly 1 million apprehensions at the southwest US border with Mexico in 2019, which represents an increase of almost half a million people compared to the prior year [4]. Since its implementation in 2019, the US Migration Protection Protocols – better known as the “Remain in Mexico” program – has mandated that asylum seekers must wait outside of the US for the duration of their immigration proceedings [5]. Consequently, there has been a substantial growth in migrant communities in towns along the Mexican border, including Tijuana, Mexicali, Ciudad Juarez, Matamoros, and Nogales. This steady influx of migrants and refugees has already strained local resources for basic necessities including food and temporary shelter. The imminent introduction of SARS-CoV-2 among members of this vulnerable population in a precarious environment has the potential to inflict devastating health consequences. Moreover, this scenario would undoubtedly overwhelm and quickly exhaust the local healthcare infrastructure in Mexican border communities.

Another important migratory crisis in the Americas is the exodus of Venezuelans fleeing political instability and a collapsed national economy. The neighboring countries hosting the largest number of Venezuelan migrants are Colombia, Brazil, Peru, and Ecuador. According to the Colombian Ministry of Foreign Affairs, there are nearly 1.8 million Venezuelans living in Colombia, and 60% of them have an “irregular” immigration status. Excluding Bogotá, Colombia’s capital city, the largest concentration of recent migrants is in the department of North of Santander, which borders the western Venezuelan states of Táchira and Zulia [6]. Despite the relatively welcoming migratory policies of the Colombian government, access to healthcare remains poor for Venezuelan migrants in Cúcuta, the largest city in the North Santander department and other border towns. With the notable exception of MedGlobal, an international non-governmental organization that is operating primary care and pre-natal clinics for Venezuelan refugees, very few philanthropic organizations are currently operating in Cúcuta to improve the substandard living conditions for Venezuelan migrants. Further complicating this difficult situation is the closure of businesses and broader decrease in economic activity during the current COVID-19 crisis. One unintended consequence of this crisis is the return of large numbers of Venezuelans back to their homeland. This abrupt movement of people across borders in the midst of a pandemic risks further complicating the COVID-19 outbreak in Venezuela, which already has a deteriorated health infrastructure.

The third important migratory current in the Americas is on the island of Hispaniola. The Dominican Republic and Haiti share the island and a complex history. Their divergent socioeconomic trajectories have resulted in an incremental eastern migration of Haitians seeking work and a better life in the Dominican Republic. Approximately 750,000 Haitians migrants and their descendants reside in the Dominican Republic, where they work primarily in agriculture and in the construction industry [7]. Historically, early waves of migrants settled and worked in the sugar cane plantations of the eastern region of the Dominican Republic. They have traditionally inhabited impromptu communities, the *Bateyes*, which have progressively grown larger and exceeded the anticipated and appropriate population sizes for which they were designed. The majority of residents of these communities live in indigence, and hence, are especially vulnerable to poverty-related diseases. Although migration patterns have changed and contemporary waves of migrants have established residence in urban areas, the issues of poverty and vulnerability to disease remain unchanged. The overcrowded living conditions and poor access to healthcare among Haitian migrants make them particularly susceptible to a rapid spread of COVID-19, which would certainly challenge the capacity of the Dominican health system to respond to the epidemic. Moreover, because Haitian migrants move regularly across the border, they may contribute to fuel transmission of the SARS-CoV-2 virus to border towns in both countries.

Considering the grave risk COVID-19 poses to these three migrant populations, it is imperative that strategies are developed and implemented now to mitigate this future potential humanitarian and healthcare system crisis. Only through proactive measures can the health and well-being of migrants be protected as well as the healthcare services of host countries be sustained. The following recommendations are proposed to address these twin goals.

**Provide international technical assistance to countries’ Ministries of Health.** The World Health Organization and its regional branch, the Pan-American Health Organization, should continue to partner with governments to facilitate expedited COVID-19 related training and capacity building of health providers and frontline workers in refugee camps, shelters, and community-based organizations.

**Increase funding to host communities and organizations working with migrants and refugees.** Wealthy nations must increase funding to relevant agencies of the United Nations Network on Migration, local community health organizations and local health departments to promptly expand preventive hygiene programs along with detection, isolation, and prompt treatment of COVID-19 cases in shelters and community based clinics. These strategies should include the building of temporary hospitals at host government furnished spaces. Where applicable, this funding could be obtained by temporarily redirecting resources from other country aid programs.

**Host countries should consider establishing non-custodial alternatives to refugee and migrant detention** [8]. This will prevent transmission of the virus in detention centers and avoid unnecessary illnesses and deaths.

**Host countries should consider temporarily halting migrant deportations to prevent the spread of COVID-19 across borders.**

**Foster public-private-academic partnerships.** Host governments should promptly identify local private and academic partners interested in working with migrants and refugees to secure private donations of funds and personal protective equipment and to take advantage of the professional expertise and ingenuity of faculty and students in areas relevant to the pandemic response.

**Promote collaboration with host countries’ professional diaspora.** Latin American countries have an enormous talent pool working in more wealthy nations who are experts in fields relevant to the pandemic response. There is eagerness by this diaspora to volunteer time and expertise to assist with refugee response. Host countries should seize this opportunity by leveraging their skills and coordinating their efforts in the campaign against COVID-19.

In addition to deciding, formulating, and implementing COVID-19 mitigation strategies in their own countries, all governments and its citizens are being confronted with a profound moral question. Namely, are the fate of migrants and refugees solely the responsibility of host nations? The answer is no. Refugees and displaced migrants are the world’s collective responsibility as unanimously agreed at the United Nations General Assembly’s New York Declaration, which recognized that “No one state can manage such movements [of refugees and migrants] on its own” and that “greater international cooperation is needed to assist host countries and communities [9].” Moreover, it would be imprudent for wealthy countries to abdicate their obligations in this time of need and displace the care of migrants and refugees to resource constrained developing nations. Not only would such a decision result in a humanitarian crisis, but also it would contribute to further sociopolitical unrest with far reaching implications in the hemisphere.
